# No evidence for inequity aversion in non-human animals: a meta-analysis of accept/reject paradigms

**DOI:** 10.1098/rspb.2024.1452

**Published:** 2024-11-27

**Authors:** Oded Ritov, Christoph J. Völter, Nichola J. Raihani, Jan M. Engelmann

**Affiliations:** ^1^ Department of Psychology, University of California, Berkeley, CA, USA; ^2^ Messerli Research Institute, University of Veterinary Medicine Vienna, Medical University of Vienna, University of Vienna, Vienna, Austria; ^3^ Department of Comparative Cultural Psychology, Max Planck Institute for Evolutionary Anthropology, Leipzig, Germany; ^4^ Department of Experimental Psychology, University College London, London, UK; ^5^ School of Psychology, University of Auckland, Auckland, New Zealand

**Keywords:** meta-analysis, fairness, inequity aversion, cooperation

## Abstract

Disadvantageous inequity aversion (IA), a negative response to receiving less than others, is a key building block of the human sense of fairness. While some theorize that IA is shared by species across the animal kingdom, others argue that it is an exclusively human evolutionary adaptation to the selective pressures of cooperation among non-kin. Essential to this debate is the empirical question of whether non-human animals are averse towards unequal resource distributions. Over the past two decades, researchers have reported that individuals from a wide range of taxa exhibit IA; tasks where participants can reject or accept a given distribution of rewards delivered the bulk of this evidence. Yet these results have been questioned on both conceptual and empirical grounds. In the largest empirical investigation of non-human IA to date, we synthesize the primary data from 23 studies using accept/reject tasks, covering 60 430 observations of 18 species. We find no evidence for IA in non-human animals in these tasks. This finding held across all species in the dataset and pre-registered subsets (all species reported to exhibit IA, primates reported to exhibit IA, chimpanzees and capuchin monkeys). Alternative interpretations of the data and implications for the evolution of fairness are discussed.

## 1. Introduction

Humans possess a sense of fairness: a set of emotional, cognitive and behavioural responses to the violation of norms governing the distribution of resources [1–6]. While the precise definition remains contested, a sense of fairness is often thought to include an aversion to unequal distribution of resources (inequity aversion [IA]), normative judgments of how resources should be distributed, and the willingness to enforce these norms. IA is thought to represent the central psychological element of the sense of fairness [2,7,8]. An individual is inequity averse when they object to inequitable resource distributions that either favour others over self (‘disadvantageous IA’) or self over others (‘advantageous IA’). IA is the result of social comparison processes that track the relative distributions of resources. For example, a child protesting the fancier gift given to their sibling is inequity averse insofar as their protest is grounded in social comparison. While the sense of fairness manifests differently across cultural contexts [9,10], IA appears to be a robust feature of human sociality [11]. In every culture studied to date, children as young as 4 years old incur costs to reject distributions that disadvantage them (thus exhibiting disadvantageous IA [7,12]). Furthermore, in resource allocation contexts, a preference for equal distributions emerges by middle childhood and persists into adulthood [13–15]. This apparent universality of disadvantageous IA in humans raises the possibility that our sense of fairness originates from an evolutionary adaptation.

IA is thought to play a pivotal role in stabilizing cooperation [13,16,17]. To establish and maintain mutually beneficial cooperation, collaborators must solve two key challenges: the generation and distribution of benefits [18]. IA may have evolved as a proximal mechanism addressing the latter, by guiding the distribution of generated benefits among collaborating partners. Inequity concerns typically manifest in contexts in which agents hold conflicting cooperative and selfish motives. For example, following a successful collaborative hunt, each hunter’s pay-offs depend on their ability to maximize their own reward while simultaneously maintaining a collaborative relationship with their partners (who in turn aim to maximize their own rewards). IA allows agents to strike a balance between these mixed motives, representing a ‘cooperativization of competition’ [19]. From an evolutionary perspective, IA may thus function to stabilize cooperative relationships in the face of competing interests. Humans are not the only species in which individuals depend on stable patterns of cooperation for survival and reproduction [20–24]. This raises the question: is IA shared with other animals?

### (a) Previous empirical work

The year 2003 marked a transition from more than 2000 years of philosophizing about the evolutionary roots of fairness (Aristotle believed that animals lack fairness because they lack speech [25]) to empirically investigating whether non-human animals show IA. In what has become one of the most famous studies (and videos [26]) of animal behaviour, dyads of capuchin monkeys participated in a token exchange task. An experimenter first handed a piece of cucumber to one monkey in exchange for the token. The experimenter then repeated this procedure with the second monkey, but, instead of a piece of cucumber, handed over a grape—a capuchin delight—in exchange for the token. Disadvantaged capuchin monkeys react in ways that suggest dissatisfaction with the inequitable treatment: they protest by failing to consume the cucumber or throwing it at the experimenter, and in some cases even refuse to participate in the task altogether. Quantitatively, a reduced willingness to exchange tokens in these inequitable conditions (compared with equitable conditions, in which both monkeys receive the same cucumber reward) was taken as evidence that the monkeys display disadvantageous IA (hereafter 'the IA hypothesis' [27]).

This publication spawned attempts to replicate and extend its findings. Using accept/reject tasks (such as the token exchange paradigm described above) and other procedures, many studies raised the possibility of IA across a wide range of taxa. Corvids [28], parrots [29], mice [30], rats [31], dogs [32], marmosets [33], tamarins [34] and chimpanzees [35–37], among other species, have all been shown to exhibit IA in at least some studies. Nevertheless, several studies did not replicate these findings [37–42], and both the robustness of the effect and the validity of its interpretation have been questioned [6,43,44].

The central point of contention is whether subjects’ responses are the result of social comparison: are subjects rejecting the lower-value food specifically because a partner is receiving higher-value food and thus exhibiting IA? Or are subjects simply disappointed because they received a lower-value food than they were expecting, regardless of what the partner is getting?

The IA hypothesis maintains that increased rejection rates in inequitable conditions are grounded in social comparison: the subject sees a partner receiving a better reward and protests the disparity by refusing to participate in the task. The main rival hypothesis (hereafter 'the disappointment hypothesis') suggests that observed rejection behaviours are the result of disappointment driven by mechanisms unrelated to social comparison. According to this view, subjects are not responding to the inequity. Rather, they experience frustration that they are not getting the more valuable reward that is presented to them; with their attention drawn to a higher-value reward, the lower-value reward becomes less palatable (also referred to as ‘individual contrast effects’ [38,39,45,46]).

The key difference between the IA hypothesis and the disappointment hypothesis is that only the IA hypothesis posits a specific effect of social comparison. Empirically, in the context of the paradigmatic IA studies, social comparison should manifest in higher rejection rates when the partner receives a higher-value reward than the subject—compared with conditions in which the experimenter merely draws the subject’s attention to a higher-value reward (without handing it to a partner), inducing disappointment without creating inequity. The importance of this distinction has been acknowledged [8], and most studies investigating IA in animals included conditions designed specifically to rule out the disappointment hypothesis. Typical examples of these are conditions in which the experimenter hands a high-value reward to an empty cage [27,44,47,48] and conditions in which the experimenter holds up a high-value reward prior to exchanging but then hands both subject and partner a low-value reward [49–55]. Some studies demonstrated an effect of social comparison above and beyond disappointment [27,35,37,52,56], while others did not [37,44,47]. Elsewhere, the difference in rejection rates between equitable and inequitable conditions did not replicate at all [37–42].

Theoretically, even increased rejection rates in social conditions could be explained through disappointment: the partner consuming a high-value reward can increase its salience to the subject, leading the subject to reject a lower-value alternative. However, such a condition difference would at least be consistent with an effect of social comparison above and beyond disappointment effects. Conversely, the absence of a difference between conditions with and without a partner receiving preferential treatment would undermine the role of a social comparison-based mechanism in these tasks. Importantly, studies using equivalent procedures in humans have found an effect of social comparison over and above disappointment effects [57,58], suggesting that a distinct, social comparison-based mechanism underlies behavioural responses to unequal allocations in humans.

### (b) The present meta-analysis

Animal behaviour studies generally use small samples [59]. Over the past decade, the field of psychology has experienced a replication crisis, which has spotlighted the need to re-evaluate key findings [60,61]. A particularly valuable tool for estimating the strength of the evidence in a field is meta-analysis [62]. The plurality of small-sample studies using comparable methodologies and equivalent outcome measures makes the question of IA in animals well-suited for meta-analytic investigation. The present study is an individual participant data (IPD) meta-analysis investigating IA in accept/reject tasks in most non-human species studied to date. In IPD meta-analyses, researchers collect the primary, participant-level data of the original studies and draw new inferences from the combined dataset (rather than synthesizing effect size estimates, as in conventional meta-analytic approaches). This approach is considered the ‘gold standard’ of meta-analysis [63,64]. Since many of the original studies used non-parametric tests, an IPD meta-analysis is particularly suited to the present question. To allow a meaningful comparison of results, we focused our investigation on studies using accept/reject tasks, the most commonly used experimental paradigm to investigate IA in non-humans. Accept/reject tasks involve one subject and one partner and report a binary outcome measure: the subject’s acceptance or rejection of an offered distribution. Researchers have also used other paradigms to test for IA in non-humans, including the choice between distributions or attitudes towards fair and unfair experimenters. Our analyses cannot speak to their results [8,65]. To our estimation, there are too few studies using other paradigms to warrant their meta-analysis at this time.

We contacted the authors of 30 eligible publications and received data for 23 of them, covering 18 species and 60 430 observations (see §2 for details and §3 for the PRISMA flow diagram of the selection process). To analyse the data, we used a series of pre-registered and exploratory mixed-effects models, which represent the key experimental features hypothesized to elicit IA while allowing generalization across studies. The models’ random effects structures allow us to account for the variability between and within species, studies and subjects, thereby providing a stringent test of the population-level effect. Using this approach, we were able to test the two key hypotheses that have been developed to account for subjects’ responses in accept/reject tasks: the IA hypothesis and the disappointment hypothesis.

The IA hypothesis and the disappointment hypothesis make divergent empirical predictions. The IA hypothesis predicts higher rejection rates only when a partner is treated better than the subject; the disappointment hypothesis predicts higher rejection rates when a higher value reward is presented, regardless of how the partner is treated. The goals of this meta-analysis are as follows: first, to test the statistical robustness of the evidence in favour of the IA hypothesis (see prediction 1 below); and second, to examine how well the disappointment hypothesis fits the data (see predictions 2 and 3 below). To account for species-level differences, we included by-species random effects in all models. Furthermore, as pre-registered, we ran each model separately for (i) all species in the dataset, as well as (ii) all species reported to exhibit IA, (iii) primate species reported to exhibit IA, (iv) chimpanzees, and (v) capuchin monkeys. The analyses reported in this meta-analysis were designed to test the following three predictions:

— *Prediction 1*: subjects will be more likely to reject a reward when a partner receives preferential treatment (this represents the main goal of the meta-analysis: testing the replicability of the IA hypothesis).
— *Prediction 2:* subjects will be more likely to reject a reward when their attention is drawn to a higher-value reward (testing the replicability of the disappointment hypothesis).
— *Prediction 3*: when a partner receives a higher-value reward than the subject, the subject will increase rejection rates more than in conditions where the higher-value reward is merely presented but not given to the partner (test of whether social comparison drives rejections above and beyond other forms of disappointment).

## 2. Methods

### (a) Inclusion criteria

#### (i) Study type and design

Experimental studies that include a manipulation designed to induce disadvantageous IA in the context of a dyadic interaction in an accept–reject paradigm were included. We did not include studies in which IA was investigated in a group context (e.g. [66]).

#### (ii) Species

All non-human species were included.

#### (iii) Manipulation

For a study to be included, the manipulation must have created clear contrasting conditions where IA behaviour was either hypothesized to occur or not. This could be operationalized in one of the following three ways: (i) equal versus unequal reward distribution (‘reward inequity’—subject receives low-value food while partner receives high-value food); (ii) equal versus unequal effort (‘effort inequity’—subject receives the same value food as the partner, but has to carry out a more effortful task); (iii) presence versus absence of a partner given an unequal reward distribution (another operationalization of reward inequity). Only conditions in which the subject received some reward in each trial and studies that included such conditions, were included. In studies that vary the value of the reward received by the subject, only low-value reward conditions were included.

#### (iv) Outcome measures

Only studies with a binary behavioural outcome variable that corresponds to whether the subject accepted an offered distribution were included. We did not include studies with non-binary outcome measures (such as much of the literature about IA in dogs, in which the outcome variable is the number of times the subject carried out a command [65]), since these data would not have been comparable with the paradigmatic IA studies included in our dataset. We also excluded studies with other types of binary outcome variables, such as choice of fair versus unfair experimenter (e.g. [67]), choice between two distributions (e.g. [31]), neuronal signals (e.g. [68]) or cooperative behaviour (e.g. [69]).

### (b) Search strategy and study selection

We conducted our bibliographic searches in March 2021 on Web of Science and PubMed, for papers published from 2003 onwards (the year the first study reporting the existence of IA in animals was published [27]), using the following queries:

Web of Science: ALL = ((fairness OR inequity OR inequality) AND (animals OR ‘non-human’ OR primates OR dogs OR monkeys OR birds))

PubMed: inequity aversion.

Duplicates were removed using EndNote [70], after which the entries were imported into Covidence [71] for screening. Two relevant papers were published after the search was conducted and screened when their authors notified us of their existence [47,53]. The first author undertook the preliminary title and abstract screening; the first and fourth authors both conducted full-text reviews of papers identified as potentially eligible.

### (c) Data collection

The primary data of two eligible papers was available online [44,51]. We contacted the authors of the remaining papers requesting access to the primary data of the relevant conditions (and explaining the goal of the study). We sent all authors our first request in April 2021, with the initial goal of ending data collection by September 2021. Since some authors required additional time to prepare the data, we extended this deadline to November 2021. We have not received any additional datasets between November 2021 and the submission of this meta-analysis.

### (d) Variable coding

#### (i) Outcome variable

*Rejection:* in typical token exchange paradigms, which represent 20 out of 23 studies included in this meta-analysis, a rejection could occur when the subject either (i) failed to provide the token to the experimenter or (ii) did not accept the offered reward. Accordingly, we coded the variable ‘rejection’ as 1 in either of those cases and 0 otherwise. In tray-pulling and spoon-holding paradigms, the variable ‘rejection’ was coded as 1 for any trial in which the subject did not carry out the task or did not accept the reward, and 0 otherwise.

#### (ii) Predictor variables

To generalize across studies, we coded two new predictor variables, which correspond to the IA hypothesis and the disappointment hypothesis, respectively.

*IA condition:* for IA to emerge, a trial should include a partner receiving a higher-value reward (‘reward inequity’) or the same reward for less effort (‘effort inequity’). Therefore, corresponding to the IA hypothesis, we coded the variable 'IA condition' as 1 when a partner was present and received a higher-value reward/the same reward for less effort, and 0 otherwise.

*Disappointment condition:* for disappointment to emerge, a higher-value reward than the one the subject is offered must be saliently presented in the trial. Therefore, we coded the variable disappointment condition as 1 for conditions in which a higher-value reward was either (i) handled by the human experimenter, whether or not it was given to a partner; or (ii) consumed by the partner without human intervention (relevant to conditions where the reward was dispensed by a machine); and 0 otherwise. Note that every condition with inequity may also induce disappointment, but the reverse is not the case. For example, many studies included conditions in which both the subject and partner were shown a high-value reward prior to exchanging but given a low-value reward after returning the token. Since both participants are treated the same, these conditions are not inequitable, but the presentation of a higher-value reward may trigger disappointment. In such conditions, IA condition was coded as 0 and disappointment condition as 1 (see electronic supplementary material, table S1 for a full breakdown of all conditions in the dataset and their respective coding).

**Table 1.** Studies included in the combined dataset.

study	species	*N*	task	IA found?
Bräuer *et al.* [40]	bonobos	5	token exchange	no
	chimpanzees	6		no
	orangutans	4		no
Brosnan & de Waal [27]	capuchins	5	token exchange	yes (only females were studied)
Brosnan *et al*. [35]	chimpanzees	20	token exchange	yes
Brosnan *et al*. [36]	chimpanzees	16	token exchange	only in males; combined analysis of both sexes not reported
Brosnan *et al*. [49]	orangutans	5	token exchange	no
Brosnan *et al*. [37]	chimpanzees	24	token exchange	no
Brosnan *et al*. [56]	rhesus macaques	20	token exchange	yes
Engelmann *et al*. [44]	chimpanzees	9	token exchange	no
Freeman *et al*. [50]	marmosets	10	token exchange	no
	owl monkeys	8		no
	squirrel monkeys	14		no
Heaney *et al*. [51]	kea	4	token exchange	no
Hopper *et al*. [52]	chimpanzees	18	token exchange	only in females; combined analysis of both sexes n.s.
Krasheninnikova *et al*. [48]	grey parrot	8	token exchange	no
	blue-throated macaw	6		no
	blue-headed macaw	6		no
	great green macaw	8		no
Laumer *et al*. [29]	Goffin’s cockatoos	9	token exchange	only effort inequity
Massen *et al*. [78]	long-tailed macaques	15	tray pulling	only with moderate effort
McAuliffe *et al.* [42]	capuchins	6	button pressing	no
Silberberg *et al*. [38]	capuchins	7	token exchange	no
Sosnowski *et al*. [53]	gorillas	8	token exchange	no
Talbot *et al*. [54]	squirrel monkeys	24	token exchange	no
Talbot *et al*. [79]	capuchins	15	token exchange	only when the subject received low-value reward; analysis combining effort levels not reported
Titchener *et al*. [47]	long-tailed macaques	12	token exchange	no
van Wolkenten *et al*. [55]	capuchins	13	token exchange	yes
Wascher & Bugnyar [28]	crows	6	token exchange	yes
	ravens	4		
Yasue *et al*. [33]	marmosets	6	spoon holding	yes

*Note*. ‘Token exchange’ refers to the token exchange paradigm. In ‘tray-pulling’ tasks, subjects need to pull a rope to make a tray with the reward accessible. In the ‘spoon-holding’ task, subjects held a spoon for 2 s before being handed a reward. In the ‘button pressing’ task, subjects pushed one of two buttons on an apparatus to accept or reject a food distribution. Figure 2*a* offers a descriptive overview of the distribution of mean between-condition differences in individual rejections in favour of the IA and disappointment hypotheses.

### (e) Data analysis

To test our hypotheses, we compared the full models with a respective null model lacking the test predictors but maintaining the same random effects structures using a likelihood ratio test. All reported *p*-values are two-tailed, with results considered significant at *p* < 0.05.

#### (i) Models

All models are mixed-effects logistic regressions that predict the likelihood of a rejection in a given trial based on characteristics of the experimental condition. To address the fact that both IA and disappointment effects are likely to operate differently in different species, we included a random intercept for species as well as within-species random slopes. The models therefore account for between-species differences in both overall rejection rates and the effect of the experimental manipulations. Similarly, we also included random intercepts and slopes for the study and subject. As pre-registered, we ran each model separately for (i) all species included in the dataset; (ii) all species reported to exhibit IA; (iii) all primate species reported to exhibit IA; (iv) chimpanzees; and (v) capuchin monkeys (see electronic supplementary material, note 1 for a list of species included in each category). These subsets of the data were included to avoid a population-level effect in some species being ‘washed out’ by species never hypothesized to exhibit IA and to assess the state of the evidence concerning the two species tested most often for IA (chimpanzees and capuchins). For models (iv) and (v), we removed the random terms for species.

It has been suggested that only species in which individuals routinely cooperate with non-kin should exhibit IA (the ‘cooperation hypothesis’ [72]). We chose to subset the species based on the reported rather than hypothesized existence of IA since only the former provides a clearly defined criterion (the cooperation hypothesis predicts that IA should only emerge in cooperative species, but not what level of cooperation suffices). For instance, while gorillas do cooperate with non-kin, it has been argued that their failure to exhibit IA supports the cooperation hypothesis since they are less cooperative than chimpanzees [53]. Furthermore, since the cooperation hypothesis is based on the empirical findings included in this meta-analysis [50], it is unclear whether one can meaningfully distinguish between species that would be hypothesized to exhibit IA according to this theory and those that have been reported to do so.

Participation in an effortful task, such as exchanging a token for a food reward, has been claimed to be a necessary pre-condition for the emergence of IA [8]. Therefore, in the pre-registration, we intended to explicitly examine the effect of whether the subject had to carry out a task—as opposed to simply being handed a reward—on rejection rates, as well as the interaction between task and condition type. However, since only one of the studies in our dataset included both equitable and inequitable conditions without a task, obtaining a reliable estimate of the interaction effect would have been impossible. Therefore, in all analyses reported below, we only included conditions in which the subject had to carry out a task.

##### Model 1 (pre-registered): does preferential treatment of a conspecific increase rejection rates in IA paradigms?

Our first model offers a straightforward test of the statistical robustness of the IA effect. According to the IA hypothesis, rejection rates should be higher in inequitable conditions. As described in §2d(ii), the variable ‘IA condition’ corresponds to whether the condition creates inequity between subject and partner and therefore serves as the predictor variable.

Model 1, which predicts the likelihood of a rejection in a given trial, took the following form:


rejection∼IAcondition+(IAcondition∣species)+(IAcondition∣study)+(IAcondition∣subject).

This model (like all others in the paper) is a mixed-effects logistic regression. The model estimates the fixed effect of IA condition on rejection rates. The variables in brackets describe the random effects structure, which includes random intercepts and slopes for species, study and subject. Thus, unlike a traditional logistic regression, this model (like all others in this paper) does not assume a uniform effect across these subpopulations [73].

##### Model 2 (exploratory): does the presentation of a higher-value reward increase rejection rates in IA paradigms?

As an exploratory follow-up analysis, we examined whether the salient presentation of a higher-value reward than the one offered to the subject increases the likelihood of a rejection. Thus, we compared conditions in which a high-value reward was either handled by a human experimenter or consumed by a partner with conditions in which a higher-value reward was neither handled by a human experimenter nor consumed by a partner (see §2d(ii) and electronic supplementary material, table S1 for a breakdown of all conditions and their coding). Inequity conditions are included, since they may cause increased rejection rates because the experimenter handles a high-value reward before giving it to a partner, causing the subject to experience disappointment at a lower-value reward. To test for the effects of disappointment, we modified model 1 by replacing IA condition with disappointment condition as the predictor:


rejection∼disappointmentcondition+(disappointmentcondition∣species)+(disappointmentcondition∣study)+(disappointmentcondition∣subject).

##### Model 3 (exploratory): does a partner receiving a higher-value reward than the subject increase rejection rates over and above the mere presentation of a higher-value reward?

For our final exploratory model, we investigated whether the existence of reward/effort inequity increases the likelihood of a rejection over and above food disappointment. To this end, we ran the same model as in model 1 but only included conditions in which a higher-value reward was either handled by the experimenter or given to a conspecific (i.e. the subset of the dataset where disappointment condition = 1). Thus, this model isolates the effect of social comparison on rejection rates: we only included conditions in which a higher-value reward was saliently presented and tested whether the fact that a social partner received this higher-value reward—or the same low-value reward for less effort—increased rejection rates. Furthermore, to avoid confounding by study, we only included studies with conditions that induced food disappointment without inequity (all studies had conditions inducing food disappointment and inequity).

### (g) Open science practices

Apart from analyses marked as exploratory, search strategy, eligibility criteria, variable coding, models and inferential criteria were pre-registered and made available online prior to data collection (https://osf.io/q8ajw?view_only=c92fddf749204f4789286006a758edda) in accordance with PRISMA-IPD reporting guidelines [64]. Unless stated otherwise, analyses conform to the pre-registration. All analyses were carried out in R [74] using RStudio [75]. We used the tidyverse packages for data wrangling and visualization [76]. Generalized linear mixed-effects models were run using the lme4 package [73]. The code used to run the analyses, as well as all model output files, are publicly available at Zenodo 77. The full datasets have not been made available since we have not received permission from the authors of the included papers to publish their data. Datasets without the outcome variable are accessible in the Zenodo repository.

## 3. Results

### (a) Data

**Figure 1.**
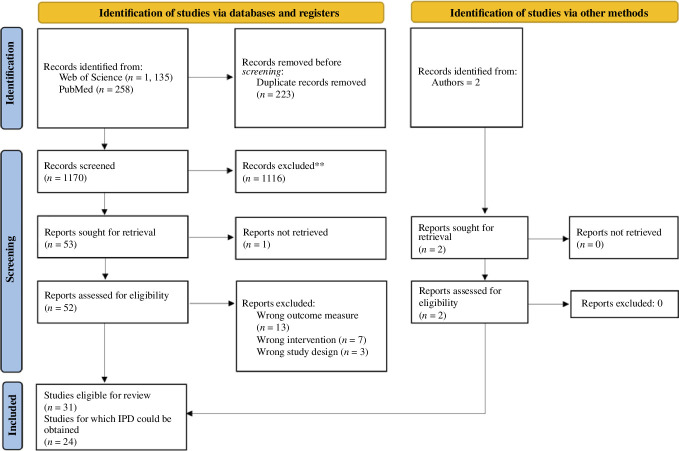
PRISMA flow diagram for new systematic reviews. Papers excluded prior to retrieval were deemed irrelevant based on title and abstract screening. Wrong outcome measures included non-binary outcome variables and behavioural measures not corresponding to acceptance of a distribution. Wrong intervention included studies that investigated IA in nondyadic (e.g. group) settings and studies that included collaborative tasks. One paper was found eligible according to our pre-registered criteria but excluded from the analyses following our decision to simplify the models and only include conditions in which the subject had to carry out a task, leading to the final count of 23 papers included in the dataset.

We identified 29 eligible papers in the literature search (see figure 1 for a PRISMA flow diagram of the selection process). Two papers were published after we conducted the literature search and added after their publication was brought to our attention [47,53]; one of these [47] was initially included as a pre-print but has since been published in a peer-reviewed journal. After requesting primary data from the authors of the eligible papers, we obtained the data for 24 studies. The decision to include only conditions with an effortful task, as explained in §2, resulted in the exclusion of one out of these 24 [41]. The final dataset included 18 species, 302 subjects and 60 430 observations. The included papers are listed in table 1. The number of subjects and studies per species is summarized in electronic supplementary material, table S2.

**Figure 2.**
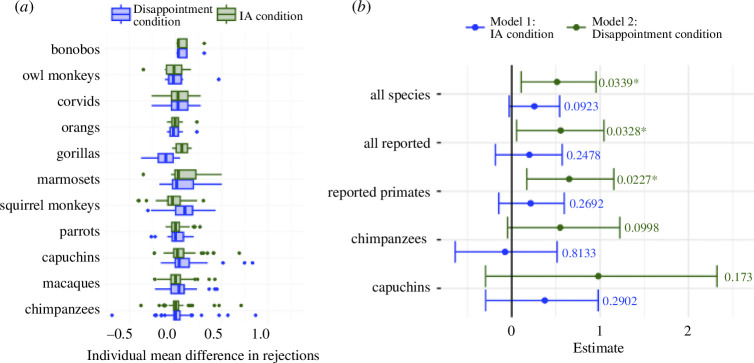
Individual differences in mean rejection rates for IA condition and disappointment condition and model estimates for models 1 and 2. (*a*) The distribution of the per-individual mean differences in rejections between conditions hypothesized and not hypothesized to prompt rejections according to IA/disappointment hypotheses. Numbers above 0 thus speak descriptively in favour of the respective hypothesis. For example, a participant who rejected all rewards in inequitable conditions and no rewards in equitable conditions would receive the value 1 for IA condition. For each subject, we calculated the difference in mean rejection rates between condition types for IA condition and disappointment condition. Thus, for IA condition, for example, each data point is the mean rejection rate for IA condition = 1 (conditions hypothesized to elicit IA) subtracted by the mean rejection rate for IA condition = 0 (conditions not hypothesized to elicit IA) for a particular subject. For the purpose of this representation (but not the analyses), different parrot, macaque and corvid species were grouped together. Species are ordered by sample size. Only conditions in which the subject had to carry out a task were included. Boxes extend from first to third quartile, with the vertical line representing the median; whiskers represent quartile ±1.5× interquartile range; points represent outlier subjects. (*b*) Dots represent log-scale model coefficient estimates for fixed effects (IA condition for model 1 in blue, disappointment condition for model 2 in green). Error bars represent bootstrapped 95% confidence intervals. *p*-values above error bars refer to full–null model comparison, with asterisk denoting statitical significance at *p* < 0.05 (see §2b).

### (b) Model 1

The IA effect was not significant for any of the species or species combinations investigated (table 2 and figure 2*a*), though *p* = 0.09 for the model investigating all species. We found no significant effects in any subsets of the data (table 2). Equivalent models investigating only reward inequity and only effort inequity are reported in electronic supplementary material, tables S3 and S4 and reveal no effect.

**Table 2.** Overview of results from models 1–3.

model	population	*K*	*N*	*β*	*p* (full–null comparison)
1	all species	23	302	0.257 (−0.029; 0.543)	*χ*^2^ (1) = 2.83, *p* = 0.0923
	IA reported species	18	204	0.200 (−0.185; 0.571)	*χ*^2^ (1) = 1.34, *p* = 0.2478
	IA reported primates	16	5	0.215 (−0.147; 0.594)	*χ*^2^ (1) = 1.22, *p* = .2692
	chimpanzees	6	81	−0.075 (−0.640; 0.514)	*χ*^2^ (1) = 0.05, *p* = 0.8133
	capuchin monkeys	5	43	0.375 (−0.294; 0.979)	*χ*^2^ (1) = 1.12, *p* = 0.2902
2	all species	23	302	0.513 (0.108; 0.955)	*χ*^2^ (1) = 4.50, *p* = 0.0339
	IA reported species	18	204	0.554 (0.055; 1.044)	*χ*^2^ (1) = 4.56, *p* = 0.0328
	IA reported primates	16	5	0.650 (0.170; 1.156)	*χ*^2^ (1) = 5.19, *p* = 0.0227
	chimpanzees	6	81	0.549 (−0.046; 1.224)	*χ*^2^ (1) = 2.71, *p* = 0.0998
	capuchin monkeys	5	43	0.980 (−0.295; 2.324)	*χ*^2^ (1) = 1.86, *p* = 0.173
3	all species	17	244	−0.001 (−0.392; 0.347)	*χ*^2^ (1) = 0, *p* = 0.992
	IA reported species	12	155	−0.019 (−0.449; 0.404)	*χ*^2^ (1) = 0.01, *p* = 0.9253
	IA reported primates	11	146	0.0431 (−0.340; 0.420)	*χ*^2^ (1) 0.05, *p* = 0.8222
	chimpanzees	5	75	−0.079 (−0.678; 0.636)	*χ*^2^ (1) = 0.05, *p* = 0.8208
	capuchin monkeys	3	30	0.332 (−0.156; 0.830)	*χ*^2^ (1) = 1.11, *p* = 0.2928

Note. *β* refers to the estimated coefficient for IA condition (models 1 and 3) and disappointment condition (model 2). *p*-value for full–null comparison corresponds to a likelihood ratio test comparing the full model with a model consisting of the same random effects structure but no fixed predictors. *n* = number of species, *K* = number of studies.

### (c) Model 2

In sub-models investigating all species, all species reported to exhibit IA, and all primate species reporting to exhibit IA, we observed a significant effect of the disappointment condition, indicating that subjects were more likely to reject a low-value reward when a higher-value reward was presented in the trial (table 2; figure 2*b*). Akaike information criterion values for models 1 and 2 are presented in electronic supplementary material, table S11. The difference scores suggest strong support for model 2 over model 1 for all populations tested.

### (d) Model 3

In all populations tested, the sub-models revealed no effect of inequity beyond that of disappointment (table 2). Equivalent models focusing on reward inequity are presented in electronic supplementary material, table S4 and show the same pattern of results.

## 4. Discussion

We investigated whether non-human animals exhibit disadvantageous IA in accept/reject tasks using an IPD meta-analysis. Based on the existing raw datasets we could obtain, we coded a new variable corresponding to whether the condition was inequitable or not (whether a partner was present and received better treatment) and used this variable as a predictor in a series of pre-registered mixed-effects logistic regressions. We found no significant effects of inequity manipulations on rejection rates. These pre-registered analyses combined two forms of IA, reward and effort inequity. Models testing each of these two forms separately (electronic supplementary material, tables S3 and S4) likewise show no significant effect. We thus find no significant evidence for the presence of IA in accept/reject tasks in non-humans. However, we did find a significant effect of food disappointment for all species in the dataset, all species reported to exhibit IA and all primate species reported to exhibit IA (in the models investigating specifically chimpanzees and capuchin monkeys, effects were positive but non-significant).

These findings shed new light on our understanding of the evolution of fairness. A full-fledged sense of fairness, which involves both disadvantageous and advantageous IA, as well as cultural values and norms related to resource distributions, is generally considered to be unique to humans [80]. What remains controversial is whether non-human animals display any of the constituents and universal elements of human fairness preferences—in particular, disadvantageous IA. Our results suggest that they do not.

While every cooperative species faces the challenge of distributing the benefits of collaborative endeavours, non-humans may rely on mechanisms unrelated to fairness to solve these (such as dominance [18]). Humans may differ from other species in this respect due to strong interdependence with social partners [17] in combination with a history of selective pressures favouring high levels of cooperation [81,82]. Another possibility is that non-human animals would benefit from social comparison-based fairness, but lack the cognitive (and possibly motivational) resources to track patterns of unequal resource distributions. The social comparison mechanism proposed by the IA hypothesis is computationally demanding (more so than that posited by the disappointment hypothesis). To protest the more favourable treatment of the partner, a subject would have to keep track of how the experimenter treats both subject and partner, compare these treatments along a principle such as equality, represent the fact that the experimenter could have treated both the same and, based on these processes, infer that the experimenter wronged the subject. It is possible that at least some non-human species lack the working memory capacity and inferential abilities required for such a complex computation [83–85]—although chimpanzees do appear to be able to compare alternative possibilities [86–88], suggesting that in some taxa motivational factors might be more important.

Which processes, if not IA, might explain subjects’ rejection of low-value rewards in the included studies? Two versions of the disappointment hypothesis—the food disappointment hypothesis and the social disappointment hypothesis—both maintain that subjects’ rejections are not grounded in social comparison but rather stem from frustration at receiving a low- rather than a high-value reward. According to the food disappointment hypothesis (also referred to as ‘individual contrast effects’ [36]), subjects experience simple disappointment due to not getting the higher-value reward they were expecting. The social disappointment hypothesis proposes that rejections are fuelled by subjects’ disappointment that the human is not treating them as well as they could—regardless of what the partner is getting [44,47,87]. Notably, social disappointment effects can emerge even in studies not specifically designed to test them, whenever the human experimenter handles a higher-value reward than the one the subject receives (e.g. by serving it either to an empty cage or to a conspecific). The only conditions that can be expected to induce food disappointment but not social disappointment are those where rewards are dispensed by a machine. Since only two studies in our dataset included conditions with both human and machine distributors [44,47], we are limited in our ability to tease apart the two versions of the disappointment hypothesis. However, we can say that the data are consistent with both. Models testing the social disappointment hypothesis are reported in the electronic supplementary material, tables S5 and S6 and show the same pattern of results as that reported for the food disappointment hypothesis.

Although we find no evidence for IA in non-human species, it is possible that IA effects emerge under specific contexts or in particular individuals [8,89]. For example, time living together [35], sex [36,52], age [56] and personality dimensions such as ‘extraversion’ [37] have all been reported to predict responses to inequity. Furthermore, methodological details such as whether the subjects are side-by-side or facing each other, the hunger level of the subjects and the contrast in reward value have been cited as critical for the appearance of IA [89]. Testing the effect of different contextual, demographic or personality-related variables was beyond the scope of the current study and would require an unfeasibly large dataset. Furthermore, some of the methodological variations suggested to inhibit the appearance of IA (e.g. hunger and difference in reward value) should theoretically affect the appearance of disappointment effects as well and thus cannot explain the evidence we find in support of the disappointment hypothesis. It is also questionable to what degree methodological variations, such as side-by-side orientation, correspond to real-world features of cooperative interactions. A full breakdown of every potential mediator is beyond the scope of this article, and we refer the reader to qualitative reviews for the argument in favour of their importance [8,89].

The multi-species analyses we conducted cannot rule out that any of the individually included species do exhibit IA, as an effect in a single species could have been ‘washed out’ by other species that do not respond to inequity. However, the cooperation hypothesis predicts that IA emerges in cooperative species, not that some specific species have arbitrarily evolved a concern with inequity [54]. Thus, the null results across species previously reported to exhibit IA—all of which are cooperative—do not support the cooperation hypothesis, which has motivated much of the research on IA in non-humans. Furthermore, we also found no effect in chimpanzees and capuchin monkeys, the two (cooperative) species studied most often in this context.

Taken together, these results suggest that IA is not robustly exhibited by non-humans in the most widely used experimental paradigm. However, we cannot rule out the possibility that IA is demonstrated by some non-human species in other paradigms or in specific social and environmental contexts. It is possible that the tested species show concern with inequity in more naturalistic settings and that the paradigm included in this meta-analysis simply failed to elicit this concern.

Our meta-analysis has several important limitations. We were able to obtain the data for 23 out of 30 eligible studies. This is a relatively large share for an IPD meta-analysis but nevertheless omits several relevant studies. While we have no reason to suspect any systematic bias against the IA hypothesis in the datasets we obtained, it is possible that an even higher powered investigation would have led to different or perhaps stronger conclusions. Crucially, only one of the seven omitted studies reported a positive finding (i.e. a finding in line with the IA hypothesis, see electronic supplementary material, table S9). It is thus unlikely that the results of our meta-analysis would have qualitatively changed if we had been able to include all studies. A second limitation of the present study is that simple frustration effects (i.e. frustration that one is receiving a lower-value reward than one has previously received) have been argued to influence rejections in IA paradigms [38,39,45] and were not tested in this meta-analysis since our dataset lacks this information. Furthermore, to be able to meaningfully compare results, we limited our inclusion criteria to paradigmatic accept/reject tasks—procedures with one subject and one partner and binary acceptance measures. We therefore excluded studies using collaborative tasks (e.g. [90,91]). Since the sense of fairness functions to support cooperation [92,93], an analysis of these studies could deliver further insights into its evolutionary origins. These criteria also led to the exclusion of all canine studies, which generally use non-binary outcome measures (e.g. number of times the subject carried out a task on command before refusing to continue), and have often reported evidence for IA [67,94–96]).

The current findings set the stage for further explorations of the mechanisms underlying cooperation in non-humans. Our sense of fairness consists, largely, of expectations for how we want and expect to be treated in relation to others [8]. It is possible that individuals in other species do not form these types of social-comparison-based expectations, but nevertheless form complex expectations for how specific social partners should treat them [87]. Our understanding of the origins of fairness would benefit greatly from identifying what these expectations are and how they are formed. Fairness is a fundamental feature of human sociality; further work on the motivational, emotional and cognitive underpinnings of cooperation in other species will contribute to a greater understanding of its evolutionary history.

## Data Availability

The code used to run the analyses, as well as all model output files, are publicly available at Zenodo [77]. The full datasets have not been made available since we have not received permission from the authors of the included papers to publish their data. Datasets without the outcome variable are also accessible in the Zenodo repository. Supplementary material is available online [97].
